# The sustainability of hospital accreditation models: a cross-sectional study

**DOI:** 10.1093/intqhc/mzaf017

**Published:** 2025-02-20

**Authors:** Mohammed Hussein, Milena Pavlova, Wim Groot

**Affiliations:** Department of Health Services Research, CAPHRI, Maastricht University Medical Center, Faculty of Health, Medicine and Life Sciences, Maastricht University, P.O. Box 616, Maastricht, Limburg 6200 MD, The Netherlands; Department of Quality and Patient Safety, Saudi German Health, P.O. Box 2616, Riyadh 13321, Saudi Arabia; Department of Health Services Research, CAPHRI, Maastricht University Medical Center, Faculty of Health, Medicine and Life Sciences, Maastricht University, P.O. Box 616, Maastricht, Limburg 6200 MD, The Netherlands; Department of Health Services Research, CAPHRI, Maastricht University Medical Center, Faculty of Health, Medicine and Life Sciences, Maastricht University, P.O. Box 616, Maastricht, Limburg 6200 MD, The Netherlands

**Keywords:** accreditation, hospital, quality improvement, sustainability, external evaluation, health policy

## Abstract

**Background:**

Despite the importance of hospital accreditation, its sustainability is jeopardized. This is due to the disparity between the rapid changes in the health sector and the accreditation standards that remain unchanged. This study aims to examine what improvements are important in enhancing the sustainability of the hospital accreditation model in Saudi Arabia.

**Methods:**

All quality managers in accredited Saudi Arabian hospitals were invited to participate in a cross‐sectional questionnaire-based study in July–August 2022. A structured questionnaire was developed, tested, piloted, and factorially validated using exploratory factor analysis. On a 5-point Likert scale, respondents were asked to rate the importance of recommended changes that are proposed to enhance the sustainability of accreditation policies, standards development, evaluation methods, and the evaluation team. The importance of the recommendations, according to the respondents, was described using the relative importance index, while multivariate linear regression was used to analyse the association with independent variables.

**Results:**

A total of 158 valid questionnaires (64% response rate) were included in the analysis. On average, participants had 6.9 (SD 2.1) years of experience in quality management. The overall mean importance attached to improving standards development, accreditation policies, evaluation team, and evaluation methods were 3.55, 3.43, 3.41, and 3.21, on a 5-point scale, respectively. Shifting the focus of accreditation standards from structure and compliance to outcomes and improvement (mean importance = 4.47), updating standards periodically to reflect current best practices and research (mean importance = 4.41), and integrating consumer perspectives in all aspects of accreditation (mean importance = 4.37) were the most important perceived recommendations. Multivariate regression analysis yielded that managers with more years of experience had significantly higher mean scores on the importance of improving accreditation policies (β = 0.120, *P *= .037), standards development (β = 0.246, *P *< .001), evaluation methods (β = 0.268, *P *< .001), and the evaluation team (β = 0.369, *P* < .001).

**Conclusions:**

Improving accreditation policies, standards development, evaluation methods, and the evaluation team are important in enhancing the sustainability of hospital accreditation programmes. This study offers insights to assist policymakers and other stakeholders in redesigning traditional accreditation models to make them more sustainable and that can supplement other performance improvement tools in improving the quality of healthcare services.

## Background

A century ago, the seeds of hospital accreditation were planted. Hospital accreditation programmes have flourished exponentially since then, and have become an important aspect of healthcare quality systems [1]. This role is not undisputed. Some have questioned the capacity of accreditation to meet the primary goal of improving quality and outcomes [2, 3]. However, recent reviews indicate that accreditation benefits are undeniable, particularly in strengthening organizational culture, standardizing practices, driving change, and increasing efficiency [4, 5]. Nevertheless, the rapid changes occurring in the health industry in an increasingly digitalized era and the enduring financial and non-financial challenges facing accreditation implementation cast doubt on the sustainability of accreditation programmes [6]. This propels accreditors to innovate and find novel channels to preserve the standing of accreditation, among other performance-improvement tools. However, the existing literature on accreditation lacks clarity on ways accreditation should evolve to demonstrate value in light of these mounting challenges [7].

Accreditation programmes share similar procedures and hurdles in various contexts [8]. However, many accreditation entities opt to work *in silos*, which may explain, in part, why the demand for change in accreditation policies has surpassed the tentative improvements made so far. In the Saudi Arabian context, over 450 hospitals provide secondary and tertiary care. A mandatory accreditation model has been adopted to supplement existing performance management tools and enhance the quality of healthcare services. The Saudi Central Board for Accreditation of Healthcare Institutions (CBAHI) is the accrediting body that establishes standards and evaluates hospital performance against them. Since its inception in 2005, CBAHI has updated accreditation standards periodically, although accreditation policies have remained unchanged. According to a recent study, the implementation of hospital accreditation standards in Saudi Arabia faces cultural, structural, and financial challenges that may jeopardize the model’s long-term viability [9]. Such challenges, in fact, were determinants of abandoning accreditation in other contexts [10, 11].

In response, the International Society for Quality in Health Care (ISQua) strives to internationalize the key pillars of accreditation programmes via sets of defined standards [12]. Additionally, experts in the field have proposed recommendations to enhance the durability of accreditation programmes, such as engaging service providers in the development of standards, incorporating artificial intelligence into accreditation evaluations, and integrating consumer perspectives in accreditation decisions [7, 8, 12, 13]. However, the importance and impact of these recommendations on sustaining accreditation have yet to be tested. Testing these recommendations may offer ostensible benefits in determining the improvements that are important for redesigning the current accreditation model. Hence, this study aims to examine the improvements that hospital quality managers believe are important to enhance the sustainability of the hospital accreditation model in Saudi Arabia.

## Methods

### Survey design

This cross‐sectional survey was conducted between July and August 2022 among the quality managers of Saudi Arabian hospitals. To prepare the questionnaire, we conducted a brief review of the accreditation literature to identify the recommended changes proposed to enhance the sustainability of hospital accreditation programmes [7, 8, 12, 13]. The literature review was conducted using PubMed, Scopus, and Web of Science, employing the search terms ‘hospital accreditation’, ‘sustainability’, and ‘recommended changes’ to ensure comprehensive coverage. In addition, the review included the ISQua Guidelines and Principles for the Development of Health and Social Care Standards [14], a key resource that provided foundational guidance for identifying relevant recommendations. In total, 70 recommendations were extracted and deduplicated, yielding 27 recommended changes in the initial draft. The research team, which possessed expertise in health policy, hospital accreditation, and quality management, iteratively refined the initial list of recommendations to ensure conciseness and precision by assessing alignment with the objectives of sustainable accreditation programmes, feasibility for implementation, and relevance to hospital settings, while excluding those deemed redundant, overly general, or lacking supporting evidence on their applicability. Based on the nature of the recommendations, we grouped them into four constructs: accreditation policies, standards development, evaluation methods, and evaluation team [15].

### Questionnaire validity

Six accreditation experts pretested the draft questionnaire for response latency, face validity, and content validity. The experts were selected based on their academic credentials and extensive experience in accreditation, holding advanced degrees (master’s or doctorate) in health-related fields, with at least 10 years of involvement in accreditation standards and evaluation practices. They received detailed instructions on how to assess the validation items for clarity and relevance.

The content validity was examined by rating each recommendation using the 4-point relevancy scale suggested by Polit et al. (1 = not relevant, 2 = somewhat relevant, 3 = quite relevant, and 4 = highly relevant) [16]. The item-level content validity index (I-CVI) was used to assess the agreement on relevancy. At the item level, the I-CVI was computed by dividing the number of those judging the recommendation as relevant (i.e. rating 3 or 4) by the total number of responses. An I-CVI cutoff of 0.83 was deemed sufficient to presume content validity, as proposed by Layn [17]. The assessment revealed four recommendations with an I-CVI below 0.83, which were subsequently eliminated, yielding 23 recommendations in the final draft. The four excluded recommendations were public reporting of accreditation status, involving methodologists in standard development, obtaining adequate evaluation samples, and matching surveyor team size to hospital capacity.

### Questionnaire construct

Following content validation, the 23 recommendations in the redesigned questionnaire were distributed as follows; accreditation policies (6 items), standards development (6 items), evaluation methods (6 items), and evaluation team (5 items). Each construct was carefully defined, and its alignment with the recommendations was verified through a consensus meeting among the research team to ensure conceptual clarity and internal consistency. In addition, the questionnaire included sociodemographic information, such as age, sex, professional background, experience, and workplace.

### Questionnaire piloting

To serve the study objective, the redesigned survey questionnaire was purposely pilot-tested on a group of 12 quality managers, which was adequate to determine feasibility [18]. The quality managers were asked to rate the importance of each recommendation on an ordinal unipolar 5-point Likert scale (1= not at all important, 5 = very important). The entire methodological approach to questionnaire administration was discussed during the piloting. The piloting results did not disclose a need for additional adjustments.

### Sampling and questionnaire administration

Quality managers in Saudi Arabian accredited hospitals were the target population because they are in charge of all accreditation phases. Assuming a 95% confidence level and 0.05 margin of error, a sample size of 151 was estimated to be adequate [19]. However, to avoid nonresponse and attrition biases, the 247 quality managers in accredited hospitals were all invited to voluntarily participate in an anonymous electronic questionnaire. The questionnaire was shared via email, using the mailing list of the national accrediting body (i.e. CBAHI). A reminder email was sent 1 week after the original email. The questionnaire was hosted electronically on SurveyMonkey (SurveyMonkey Inc., San Mateo, CA, USA).

### Data analysis

The filled-in questionnaires were reviewed for completeness, cleaned for missing values, and coded pre-analysis. As appropriate, the mean and standard deviation were used to describe continuous variables, whereas categorical variables were described as frequencies and percentages. Statistical normality and homogeneity were assessed using Kolmogorov–Smirnov and Levene’s tests, respectively. Before conducting multivariate linear regression, assumptions of linearity, normality, and homoscedasticity were verified using graphical inspection of residual plots. Multicollinearity among predictors was assessed via variance inflation factor (VIF) values, with a threshold of VIF < 5 considered acceptable. All assumptions were met, ensuring model reliability. The Spearman (rho) correlation test was used to assess correlations between the measured constructs. Furthermore, the predictors of perceived importance were assessed using multivariate linear regression analysis. For each of the four constructs, the mean perceived importance, as per the respondents, was used solely as a dependant variable to regress the perception of quality managers against their sociodemographic characteristics and workplaces. Statistical analyses were performed using IBM SPSS Statistics (version 27.0. Armonk, NY, USA). Alpha level was set at 0.05 to determine the level of significance.

Furthermore, the relative importance index (RII) analysis was used to rank the recommendations for each construct [20]. Specifically, relative importance was expressed as a percentage, in which 0–25, 25–50, 50–75, and >75% indicated very insignificant, insignificant, significant, and very significantly important, respectively. The RII was computed using the following formula, where W = the importance rate given for an item on the 5-point scale, A = maximum possible score, and *N* = s total number of samples.


$${\boldsymbol{RII}} = \mathop \sum \limits_1^n W \div A \times N$$

### Questionnaire reliability and factorial validity

The internal consistency was measured using Cronbach’s alpha test to ensure that the recommendations fit conceptually within each construct. The Cronbach’s alpha coefficients for the overall questionnaire and the four constructs (i.e. accreditation policies, standards development, evaluation methods, and the evaluation team) were 0.79, 0.84, 0.81, 0.79, and 0.86, respectively, indicating good internal consistency [21]. However, four recommendations (shown in the Supplementary file) denoted low shared covariance to the overall internal consistency, which imposes using exploratory factor analysis (EFA) to assess the factorial validity of the questionnaire and characterize the questionnaire’s items. The EFA procedures were relevant because of the nonredundant correlations between the questionnaire items, as evident by Bartlett’s test for sphericity and sufficient sampling as per the Kaplan–Meyer–Olkins index of sampling adequacy. In the EFA, the principal axis factoring method was used and preferred over other estimation methods to recover weak factors due to the ordinal unipolar nature of the scale, whereas the parallel analysis (PA) test was used to determine the number of items to retain from factor analysis. The PA test indicated that the questionnaire items were coalesced and loaded saliently under their relevant intended latent constructs, and four subtle constructs in the questionnaire were statistically justified. Therefore, the EFA results were accepted because they were simple, meaningful, theoretically sound, and could be interpreted in the context of research. The detailed EFA analysis is shown in the Supplementary file.

## Results

### Participants’ characteristics

In total, 158 quality managers participated in the study (64% response rate), exceeding the minimum required sample size, estimated to be 151. Most managers were men (66%), had a bachelor’s degree (70%), and had medicine (30%) as the most prevalent professional background. Furthermore, the mean age of the participants was 37.1 (SD 5.1) years and the majority (89%) worked in hospitals that provided acute care services. On average, participants had 6.9 (SD 2.1) years of experience in the quality management field; most (60%) had at least one credential in quality management (Table 1).

**Table 1.** Descriptive analysis of participants’ demographic characteristics (*N* = 158).

	Frequency	Percentage
Sex		
Male	104	65.8
Female	54	34.2
Educational level		
Associate/diploma degree	11	7
Bachelor’s degree	111	70.3
Higher studies (master or PhD)	36	22.8
Professional background		
Physician	48	30.4
Nurse	32	20.3
Health/hospital administration	30	19
Pharmacists	14	8.9
Technicians and auxiliary services	34	21.5
Age in years, mean (SD)		37.10 (5.1)
Years of experience in healthcare, mean (SD)		11.52 (4.74)
Years of experience in quality management, mean (SD)		6.92 (2.10)
Credentials in quality management		
Yes	95	60.1
No	63	39.9
Experience in accreditation programmes		
Only national programme	120	75.9
National and international programmes	38	24.1
Healthcare sector		
Public	106	67.1
Private	52	32.9
Hospital type		
General	141	89.2
Specialized	17	10.8

### Recommendations to enhance accreditation sustainability

On a 5-point importance scale (5 being very important), quality managers rated the importance of recommended changes that sought to enhance the sustainability of hospital accreditation constructs, accreditation policies, standards development, evaluation methods, and evaluation teams. The descriptive findings are displayed in Table 2.

**Table 2.** Descriptive and relative importance index analysis of the recommended improvements to accreditation aspects.

	Importance level (1–5)*							
Recommended improvements	Not at all important	Slightly important	Moderate important	Important	Very Important	Mean	SD	RII**
**Recommended improvements in accreditation policies *(Cronbach’s alpha = 0.84)***						**3.43**	**0.85**	
Integrating consumer perspectives in all aspects of accreditation including decisions.	6.3	6.3	3.8	10.8	72.8	4.37	1.21	87.5
Aligning accreditation standards with the requirements of other levers in the country	2.5	8.2	5.1	20.9	63.3	4.34	1.06	86.8
Automation of the accreditation process (e.g. online registration and monitoring).	7.0	10.1	10.1	38.6	34.2	3.83	1.21	76.6
Strengthen the health licensure system and other accreditation prerequisites.	10.1	19.0	37.3	14.6	19.0	3.13	1.22	62.7
Assuming unannounced accreditation visits.	3.8	34.2	36.7	9.5	15.8	2.99	1.11	59.9
Adopting a mandatory accreditation scheme.	44.3	33.5	13.3	4.4	4.4	1.91	1.07	38.2
**Recommended improvements in standards development *(Cronbach’s alpha = 0.81)***						**3.55**	**0.78**	
Shifting the focus from structure and compliance to outcomes and improvement.	1.9	6.3	8.2	10.1	73.4	4.47	1.01	89.4
Updating standards periodically to reflect current best practices and research.	3.8	3.8	9.5	13.3	69.6	4.41	1.06	88.2
Involving service users and policymakers in standard development	7.0	6.3	1.9	51.3	33.5	3.98	1.11	79.6
Adopting technically tailored standards in response to major national adverse events.	7.0	2.5	10.1	50.6	29.7	3.94	1.06	78.7
Embracing environmental-friendly standards (i.e. supporting eco-friendly guidelines)	18.4	57.6	8.2	8.9	7.0	2.28	1.08	45.7
Involving partners from outside the healthcare industry in standards development	31.6	39.2	10.8	12.7	5.7	2.22	1.19	44.3
**Recommended improvements in evaluation methods *(Cronbach’s alpha = 0.79)***						**3.21**	**0.85**	
Emerging telehealth and artificial intelligence in accreditation evaluation.	11.4	3.2	5.1	7.6	72.8	4.27	1.37	85.4
Substituting snapshot evaluation by continuous clinical performance triggers.	7.0	7.0	6.3	13.3	66.5	4.25	1.26	85.1
Integrating patient-reported outcomes into the evaluation process.	13.9	5.1	9.5	46.8	24.7	3.63	1.29	72.7
Using a combination of onsite and off-site evaluation (i.e. hybridized)	7.6	19.6	46.8	8.9	17.1	3.08	1.13	61.6
Utilizing the results of surveys (e.g. safety culture survey) to assess compliance with some aspects.	31.0	50.6	2.5	5.1	10.8	2.14	1.22	42.8
Using the tracer methodology to assess compliance	45.6	32.9	15.8	1.9	3.8	1.85	1.01	37.1
**Recommended improvements in evaluation team *(Cronbach’s alpha = 0.86)***						**3.41**	**1.02**	
Implementing strategies to minimize variations among surveyor teams	7.6	7.0	3.8	10.1	71.5	4.31	1.28	86.2
Recruiting surveyors based on robust selection criteria.	9.5	12.0	4.4	36.1	38.0	3.81	1.32	76.2
Train surveyors effectively on the required evidence of standards compliance.	13.9	5.7	2.5	46.2	31.6	3.76	1.33	75.2
Including multidisciplinary surveying team during accreditation visit.	15.2	24.7	36.1	7.0	17.1	2.86	1.26	57.2
Matching the survey team members’ expertise with the hospital’s scope of service.	24.1	48.7	8.2	10.1	8.9	2.31	1.2	46.2

* Importance level and relative importance index (RII) expressed as percentages; ** RII = $\mathop \sum \limits_1^n W \div A \times N$ (*W* = the importance rate given for an item on the 5-point scale, *A* = maximum possible score, and *N* = total number of the sample).

#### Accreditation policies

The overall mean importance of improving accreditation policies was 3.43, indicating moderate to high importance from the perspective of quality managers. Integrating consumer perspectives in all aspects of accreditation including decisions was rated the most important recommendation (mean = 4.37, RII = 87.5%), followed by ‘aligning accreditation standards with the requirements of other levers in the country’. Conversely, ‘adopting mandatory accreditation’, was perceived as insignificantly important (mean = 1.91, RII = 38.2%).

#### Standards development

Among the four constructs, the importance of improving standards development processes was rated the highest (mean = 3.55). In that, the most important perceived recommendation (mean = 4.47, RII = 89.4%) was ‘shifting the standards focus from structure and compliance to outcomes and improvement’, followed by ‘updating standards periodically to reflect current best practices and research’. In contrast, ‘involving partners from outside the healthcare industry in standards development’, was deemed insignificantly important (mean = 2.22, RII = 44.3%).

#### Evaluation methods

The perceived importance of improving accreditation evaluation methods was close to moderate (mean = 3.21). The recommendation of ‘emerging telehealth and artificial intelligence in accreditation evaluation’ was rated the highest (mean = 4.27, RII = 85.4%), followed by ‘substituting snapshot evaluation by continuous clinical performance triggers to assess compliance with standards’. However, ‘using the tracer methodology to assess compliance’ was perceived as insignificantly important and had the lowest rating of all recommendations (mean = 1.85 and RII = 37.1%).

#### Evaluation team

In the opinion of quality managers, the overall mean importance of improving the evaluation team was 3.41, denoting moderate to high importance. Of which, the recommendation regarded as the most important (mean = 4.31, RII = 86.2%) was ‘implementing strategies to minimize variations among the survey teams’, followed by ‘recruiting surveyors based on robust selection criteria’. Whereas ‘matching the survey team members’ expertise with the hospital’s scope of service’ was perceived as a substantive recommendation, but with low relative importance ratings (mean = 2.31, RII = 46.2%).

### Predictors of improving accreditation aspects

The assumptions for multiple regression analysis were tested and met. Residuals displayed linearity, normality, and homoscedasticity when inspected graphically. Multicollinearity was evaluated through VIF values for all predictors, which ranged between 1.2 and 3.6, confirming no significant multicollinearity issues (VIF < 5). Bivariate Spearman’s (rho) correlation tests revealed that managers with more years of experience in quality management tended to perceive the importance of improving the four accreditation constructs substantially higher (shown in the Supplementary file). Similarly, multivariate linear regression analysis revealed that managers with more years of experience attached significantly higher importance to improving accreditation policies *(*β = 0.120, *P*= .037), standards development (β = 0.246, *P* < .001), evaluation methods (β = 0.268, *P* < .001), and the evaluation team (β = 0.369, *P* < .001). in addition, other demographic variables converged significantly, either positively or negatively, with one or more accreditation constructs. For instance, managers with credentials in quality management predicted a significantly higher mean perceived importance in terms of evaluation methods (β = 0.365, *P* = .021), whereas those in a private workplace converged significantly but negatively with the mean perceived importance score of the evaluation team (β = −0.480, *P* = .024). Significant predictors of each construct are summarized in Table 3, and a detailed analysis is available in the Supplementary file.

**Table 3.** Multivariate linear regression analysis of the perceived importance of recommended improvements to hospital accreditation programmes*.

			95% CI for β coefficient		
Dependent variable	Predictors	Standardized β Coefficient	Lower	Upper	*P*-value
Accreditation policies, importance score ^a^	(Constant)	2.091	1.156	3.027	**<.001**
	Educational level, higher education	0.285	0.068	0.503	**.011**
	Experience in national & international accreditation	0.592	0.197	0.987	**.004**
	Years of experience in quality management	0.120	0.007	0.232	**.037**
	Perceived importance of evaluation methods	−0.184	−0.332	−0.036	**.015**
Standards development, importance score ^b^	(Constant)	2.716	1.880	3.552	**.000**
	Educational level, higher education	−0.238	−0.454	−0.022	**.031**
	Years of experience in quality management	0.246	0.142	0.350	**<.001**
	Perceived importance of evaluation team	−0.159	−0.276	−0.043	**.008**
Evaluation methods, importance score *^c^*	(Constant)	3.576	2.695	4.458	**<.001**
	Having credentials in quality management	0.365	0.055	0.675	**.021**
	Years of experience in quality management	0.268	0.154	0.381	**<.001**
	Perceived importance of accreditation policies	−0.213	−0.382	−0.044	**.014**
	Perceived importance of evaluation team	−0.258	−0.385	−0.131	**<.001**
Evaluation team, importance score *^d^*	(Constant)	4.898	3.816	5.981	**<.001**
	Hospital sector, private	−0.480	−0.895	−0.065	**.024**
	Years of experience in quality management	0.369	0.233	0.504	**<.001**
	Perceived importance of standards development	−0.255	−0.468	−0.041	**.020**
	Perceived importance of evaluation methods	−0.386	−0.574	−0.199	**<.001**

* Only predictors with *P* < .05 are shown in the table (detailed analysis is available in Appendix 1, Tables 4–7).

a Model *R* = 0.641, adjusted *R*^2^ = 0.327; ^b^Model *R* = 0.587, adjusted *R*^2^ = 0.299; ^c^Model *R* = 0.558, adjusted *R*^2^ = 0.269; ^d^Model *R* = 0.552, adjusted *R*^2^ = 0.257.

## Discussion

### Statement of principal findings

Numerous improvements have been endorsed to enhance the sustainability of accreditation. This study examined the improvements important for enhancing the sustainability of the hospital accreditation model in Saudi Arabia. Overall, the findings indicate the importance, according to hospital quality managers, of multiple improvements in accreditation policies, standards development processes, evaluation methods, and evaluation teams to enhance the sustainability and long-term relevance of the hospital accreditation scheme in Saudi Arabia. Notably, the multivariate linear regression showed that participants were more likely to perceive the importance of introducing these improvements when they had more years of experience in the quality management field. This may be due to the association between years of experience and knowledge of accreditation standards, as explained in a recent study conducted in Saudi Arabia [22].

### Interpretation within the context of the wider literature

In terms of accreditation policies, the perceived importance of integrating patient perspectives in accreditation aspects lends credence to a recent review that found no association between accreditation outcomes and patient experience [4]. In the person-centred era, accrediting bodies in Saudi Arabia and other parts of the world continue to disregard the results of patient experience surveys in the accreditation decision matrix [23], which is in contrast with the value of shared decision-making and possibly justifies why this recommendation was deemed important from the study respondents’ perspective. The findings further indicate the importance of harmonizing accreditation standards with other national health policies. This may assist in preventing conflicting requirements from different national authorities and in empowering the adoption of accreditation as an incentive tool [9]. In contrast, survey respondents viewed adopting unannounced and mandatory accreditation policies as relatively less important. This may be attributed to the mixed results on the value of these policies [24] and the additional stresses that these policies may cause on top of accreditation preparation stress, as this result reflects the viewpoint of quality managers specifically [25].

Since most standards in Saudi hospital accreditation are structure- and process-focused, the findings emphasize the importance of shifting the focus of the standards to outcome and improvement. This is consistent with the trend toward value-based healthcare [26], as well as prior studies that have linked the structural nature of standards to the uncertainty of accreditation outcomes [6]. Respondents also underscored the significance of periodically revising standards to keep abreast with evidence-based practices. Such change is fundamental, yet vital, to sustain accreditation over time. Therefore, now more than ever, accreditation standards need to be updated more often as what is acceptable and matters to patients today may not be relevant in the near future [27].

Additionally, study respondents stressed the importance of artificial intelligence in sustaining accreditation processes. This may be attributed to the power of artificial intelligence in nurturing the accreditation process with time-saving alternative solutions that could allow it to sustain itself longer. The lessons learned about how the COVID-19 pandemic has affected the conformance to accreditation activities in Saudi Arabia undoubtedly contributed to this emphasis [28]. Furthermore, respondents questioned the credibility of the snapshot accreditation evaluation [29]. Hence, they perceived the importance of substituting the typical snapshot evaluation with continuous monitoring of compliance with accreditation standards. In terms of the survey team, respondents stressed the need to implement strategies to reduce inter-surveyor variability. A previous study in the Saudi context reported this variation to be a contributing factor to stakeholder disengagement with accreditation [9]. Surprisingly, in contrast to prior studies that described tracer methodology as a helpful tool for evaluating compliance with accreditation standards in other contexts [30], this approach was regarded as the least important in terms of sustaining the Saudi Arabian hospital accreditation model in our study.

### Implications for policy, practice, and research

The study underscored several key implications for the future of hospital accreditation. For instance, integrating patient voice into accreditation policies was viewed as a critical enabler to enhance the relevance and effectiveness of accreditation outcomes. Also, from a policymaker angle, harmonizing accreditation standards with national health policies can prevent contradictory requirements and promote accreditation as a valuable incentive tool. Nonetheless, the key implications support redesigning accreditation models. Accreditors are encouraged to regularly update accreditation standards to maintain their relevance, incorporate time-saving solutions, such as artificial intelligence into accreditation processes, replace the traditional snapshot evaluation with a continuous accreditation compliance monitoring system, and shift the focus of accreditation standards to outcomes in order to further enhance the credibility and effectiveness of accreditation process. In light of these findings, it is crucial to replicate this study in different contexts and examine the perceptions of various stakeholders, including accreditation surveyors, hospital managers, and policymakers. This broader perspective will help shape the future development of accreditation models, ensuring they are robust, relevant, and responsive to the needs of all stakeholders involved.

### Strengths and limitations

The novelty of this study is its quantitative evaluation of the proposed improvements to accreditation aspects as to the importance, which, to our knowledge, has not been performed previously. The procedure employed to confirm the factorial validity of the study instrument contributed to the credibility of the findings. However, these findings should be interpreted in the context of their limitations. Despite its commonality in collecting research data, the perception-based design of the study may leave the results vulnerable to self-reporting bias. Also, the findings cannot necessarily be generalized since it was conducted in only one country and sought only the viewpoints of quality managers. Nonetheless, the evidence we arrived at is relevant to a wider context due to the international similarity of accreditation features [8].

## Conclusion

This study highlights the importance, from the perspective of the quality managers, of a set of improvements recommended to enhance the sustainability of the accreditation model in Saudi Arabia. The findings indicate the importance of introducing improvements to accreditation policies (e.g. integrating consumer perspectives in accreditation), standards development (e.g. shifting the focus of the standards to outcomes), evaluation methods (e.g. emerging artificial intelligence in accreditation evaluation), and evaluation teams (e.g. reducing inter-surveyor variability). The lessons gleaned from this study demonstrate plausible implications on a larger contextual scale. The findings can assist policymakers, accrediting entities, and other stakeholders, nationally and internationally, to better understand the ways to preserve the relevance of accreditation models, among other quality improvement tools. Hence, accrediting bodies, with involvement from experienced quality managers and relevant stakeholders, should act on these findings to produce an enhanced version of the accreditation scheme that can supplement other performance improvement tools to improve the quality of healthcare services.

## Supplementary Material

mzaf017_Supp

## Data Availability

Data underlying the findings of this study are available within the article (and/or) its supplemental materials. Additional data will be shared at reasonable request to the corresponding author in a de-identified form for confidentiality purposes.

## References

[1] Nicklin W, Fortune T, van Ostenberg P et al. Leveraging the full value and impact of accreditation. *Int J Qual Health Care* 2017;29:310–2. doi: 10.1093/intqhc/mzx010 28453825

[2] Bracewell N, Winchester DE. Accreditation in health care: does it make any difference to patient outcomes? *BMJ Qual Saf* 2021;30:845. doi: 10.1136/bmjqs-2020-012533 33542065

[3] Jha AK . Accreditation, quality, and making hospital care better. *JAMA* 2018;320:2410–1. doi: 10.1001/jama.2018.18810 30561469

[4] Hussein M, Pavlova M, Ghalwash M et al. The impact of hospital accreditation on the quality of healthcare: a systematic literature review. *BMC Health Serv Res* 2021;21:1057. doi: 10.1186/s12913-021-07097-6 PMC8493726 34610823

[5] Van Wilder A, Bruyneel L, De Ridder D et al. Is a hospital quality policy based on a triad of accreditation, public reporting and inspection evidence-based? A narrative review. *Int J Qual Health Care* 2021;33:1–7. doi: 10.1093/intqhc/mzab085 34013956

[6] Al-Alawy K, Azaad Moonesar I, Obaid HAM et al. A mixed-methods study to explore the impact of hospital accreditation. *Inquiry* 2021;58:1–8. 10.1177/0046958020981463 PMC7970682 33525936

[7] Nicklin W, Engel C, Stewart J. Accreditation in 2030. *Int J Qual Health Care* 2020;33:1–5. 10.1093/intqhc/mzaa156 33351075

[8] Greenfield D, Iqbal U, O’Connor E et al. An appraisal of healthcare accreditation agencies and programs: similarities, differences, challenges and opportunities. *Int J Qual Health Care* 2021;33:1–7. doi: 10.1093/intqhc/mzab150 34718602

[9] Hussein M, Pavlova M, Groot W. An evaluation of the driving and restraining factors affecting the implementation of hospital accreditation standards: a force field analysis. *Int J Healthc Manag* 2023;16:167–75. doi: 10.1080/20479700.2022.2084810

[10] Bogh SB, Blom A, Raben DC et al. Hospital accreditation: staff experiences and perceptions. *Int J Health Care Qual Assur* 2018;31:420–7. doi: 10.1108/IJHCQA-06-2017-0115 29865965

[11] Lane J, Barnhart S, Luboga S et al. The emergence of hospital accreditation programs in East Africa: lessons from Uganda, Kenya, and Tanzania. *Global J Med Public Health* 2014;3:1–10.

[12] Hinchcliff R . Advancing the accreditation economy: a critical reflection. *Int J Qual Health Care* 2021;33:1–2. doi: 10.1093/intqhc/mzab154 34865015

[13] Mosadeghrad AM . Hospital accreditation: the good, the bad, and the ugly. *Int J Healthc Manag* 2021;14:1597–601. doi: 10.1080/20479700.2020.1762052

[14] International Society for Quality in Health Care (ISQua) . Guidelines and Principles for the Development of Health and Social Care Standards. 5th ed. Version 1.1, ISQua; 2022. https://ieea.ch/wp-content/uploads/2024/09/Guidelines-and-Principles-for-the-Development-of-Health-and-Social-Care-Standards-5th-Edition-v1.1.pdf (10 July 2022, date last accessed).

[15] Mosadeghrad AM, Ghazanfari F. Developing a hospital accreditation model: a Delphi study. *BMC Health Serv Res* 2021;21:879. doi: 10.1186/s12913-021-06904-4 PMC8393439 34445975

[16] Polit DF, Beck CT. The content validity index: are you sure you know what’s being reported? Critique and recommendations. *Res Nurs Health* 2006;29:489–97. doi: 10.1002/nur.20147 16977646

[17] Lynn MR . Determination and quantification of content validity. *Nurs Res* 1986;35:382–5. doi: 10.1097/00006199-198611000-00017 3640358

[18] Julious SA . Sample size of 12 per group rule of thumb for a pilot study. *Pharm Stat* 2005;4:287–91. doi: 10.1002/pst.185

[19] Raosoft . *Raosoft Sample Size Calculator*. Raosoft, Inc., Seattle. 2004. (15 September 2022, date last accessed). http://www.raosoft.com/samplesize.html

[20] Holt GD . Asking questions, analyzing answers: relative importance revisited. *Constr Innov* 2014;14:2–16. doi: 10.1108/CI-06-2012-0035

[21] DeVellis RF . *Scale Development: Theory and Applications*. 4th Los Angeles: SAGE, 2017

[22] Hussein M, Pavlova M, Groot W. The attitude of hospital directors towards normalising accreditation standards: a qualitative descriptive study for Saudi Arabia. *Int J Qual Health Care* 2022;34:1–7. doi: 10.1093/intqhc/mzac070 PMC9470101 36047710

[23] Auras S, Geraedts M. Patient experience data in practice accreditation—an international comparison. *Int J Qual Health Care* 2010;22:132–9. doi: 10.1093/intqhc/mzq006 20144943

[24] Nicolaisen A, Bogh SB, Churruca K et al. Managers’ perceptions of the effects of a national mandatory accreditation program in Danish hospitals. A cross-sectional survey. *Int J Qual Health Care* 2019;31:331–7. doi: 10.1093/intqhc/mzy174 30476098

[25] Al-Faouri I, Al-Dmour A, Al-Ali N et al. Effect of Health Care Accreditation Council survey site visit on perceived stress level among Jordanian healthcare providers. *Nurs Forum* 2019;54:30–7. doi: 10.1111/nuf.12294 30508264

[26] Zanotto BS, Etges APBDS, Marcolino MAZ et al. Value-based healthcare initiatives in practice: a systematic review. *J Healthc Manag* 2021;66:340–65. 34192716 10.1097/JHM-D-20-00283 PMC8423138

[27] Mitchell JI, Graham ID, Nicklin W. The unrecognized power of health services accreditation: more than external evaluation. *Int J Qual Health Care* 2020;32:445–55. doi: 10.1093/intqhc/mzaa063 32514539

[28] El-Sherif DM, Abouzid M, Elzarif MT et al. Telehealth and artificial intelligence insights into healthcare during the COVID-19 pandemic. *Healthcare* 2022;10:385. doi: 10.3390/healthcare10020385 PMC8871559 35206998

[29] Devkaran S, O’Farrell PN, Ellahham S et al. Impact of repeated hospital accreditation surveys on quality and reliability, an 8-year interrupted time series analysis. *BMJ Open* 2019;9:e024514. doi: 10.1136/bmjopen-2018-024514 PMC6398692 30772852

[30] Vali L, Mehrolhasani MH, Mirzaei S et al. Challenges of implementing the accreditation model in military and university hospitals in Iran: a qualitative study. *BMC Health Serv Res* 2020;20:698. doi: 10.1186/s12913-020-05536-4 PMC7392663 32727444

