# Supplementary material for: The sustainability of hospital accreditation models: a cross-sectional study
Source: Int J Qual Health Care. 2025 Feb 20;37(1):mzaf017. doi: 10.1093/intqhc/mzaf017 (PMC11908374; doi:10.1093/intqhc/mzaf017)
Supplement: mzaf017_Supp [file mzaf017_supp.zip › suppl_data/Supp Materials.pdf]

Supplemenarty Material:

Table 1: The questionnaire item-corrected total statistics

Table 2: Principal Axis Factoring (PAF) Exploratory Factor Analysis Oblimin Rotated factor solution for the questionnaire items.

Table 3: Bivariate Spearman's (Rho) correlations between the perceived importance of accreditation constructs

Table 4: Multivariate linear regression analysis of the perceived importance of recommended improvements to accreditation policies.

Table 5: Multivariate linear regression analysis of the perceived importance of recommended improvements to standards development.

Table 6: Multivariate linear regression analysis of the perceived importance of recommended improvements to evaluation methods.

Table 7: Multivariate linear regression analysis of the perceived importance of recommended improvements to the evaluation team.

Table 1: The questionnaire item-corrected total statistics

	Corrected Item-Total Correlation	Cronbach's Alpha if Item Deleted
**Accreditation Policies**		
1. Aligning accreditation standards with the requirements of other levers in the country.	.368	.787
2. Adopting a mandatory accreditation scheme.	.408	.785
3. Assuming unannounced accreditation visits.	.311	.790
4. Integrating consumer perspectives in all aspects of accreditation including decisions.	.357	.788
5. Strengthen the health licensure system and other accreditation prerequisites.	.513	.779
6. Automation of the accreditation process (e.g. online registration and monitoring).	.477	.781
**Standards Development**		
7. Updating standards periodically to reflect current best practices and research.	.510	.781
8. Adopting technically tailored standards in response to major national adverse events.	.363	.788
9. Involving partners from outside the healthcare industry in standards development.	.390	.786
10. Involving service users and policymakers in standard development.	.380	.787
11. Shifting the focus of standards from structure and compliance to outcomes and improvement.	.343	.789
12. Embracing environmental-friendly standards (i.e., standards supporting eco-friendly guidelines).	.386	.787
**Evaluation Methods**		
13. Emerging telehealth and artificial intelligence in accreditation evaluation.	.395	.786
14. Using a combination of onsite and off-site evaluation (i.e. hybridized).	**.195***	.796
15. Integrating patient-reported outcomes into the evaluation process	.335	.789
16. Using the tracer methodology to assess compliance.	**.165***	.797
17. Utilizing the results of surveys (e.g. safety culture survey) to assess compliance with some aspects.	**.197***	.796
18. Substituting snapshot evaluation by continuous clinical performance triggers.	.428	.784
**Evaluation Team**		
19. Recruiting surveyors based on robust selection criteria.	**.149***	.800
20. Train surveyors effectively on the required evidence of standards compliance.	.364	.787
21. Implementing strategies to minimize variations among surveyor teams.	.300	.792
22. Including multidisciplinary surveying team during accreditation visit.	.300	.793
23. Matching the survey team members' expertise with the hospital's scope of service.	.301	.793

** corrected items indicating low shared covariance to the overall questionnaire internal consistency.*

Table 2: Principal Axis Factoring (PAF) Exploratory Factor Analysis Oblimin Rotated factor solution for the questionnaire items.

	Extracted Factors*			
	Accreditati on policies	Standards developing	Evaluation methods	Evaluation teams
Strengthen the health licensure system and other accreditation prerequisites.	.867			
Automation of accreditation process (e.g. online registration and monitoring).	.835			
Integrating consumer perspectives in all aspects of accreditation including decisions.	.653			
Aligning accreditation standards with the requirements of other levers in the country.	.573			
Adopting a mandatory accreditation scheme.	.557			
Assuming unannounced accreditation visits.	.545			
Involving service users and policymakers in standard development.		.825		
Updating standards periodically to reflect current best practices and research.		.714		
Shifting the focus of standards from structure and compliance to outcomes and improvement.		.709		
Adopting technically tailored standards in response to major national adverse events.		.597		
Embracing environmental-friendly standards (i.e., standards supporting eco-friendly guidelines).		.546		
Involving partners from outside the healthcare industry in standards development.		.429		
Substituting snapshot evaluation by continuous clinical performance triggers.			.822	
Integrating patient-reported outcomes into the evaluation process.			.765	
Using a combination of onsite and off-site evaluation (i.e. hybridized).			.659	
Emerging telehealth and artificial intelligence in accreditation evaluation.			.642	
Utilizing the results of surveys (e.g. safety culture survey) to assess compliance with some aspects.			.528	
Using the tracer methodology to assess compliance.			.503	
Train surveyors effectively on the required evidence of standards compliance.				.918
Implementing strategies to minimize variations among surveyor teams.				.775
Including multidisciplinary surveying team during accreditation visit.				.709
Recruiting surveyors based on robust selection criteria.				.694
Matching the survey team members' expertise with the hospital's scope of service.				.597

** Extraction Method: Principal Axis Factoring. Rotation Method: Oblimin with Kaiser Normalization.*

Note on *Table 2:*

As the questionnaire was constructed using various literature on hospital accreditation, the exploratory factor analysis (EFA) through the principal axis factoring (PAF) estimation method was applied to assess the factorial structure and validity of the questionnaire items. PAF was preferred over other estimation methods due to the ordinal unipolar nature of the scale. Initially, the Kaplan-Meyer-Olkins index of sampling adequacy was 0.71, Bartlett's test for sphericity showed non-redundant correlations between the questionnaire items ($\chi^2(253)=0.1755.4$, $P<0.001$), and the determinant index was 0.001. These 3 measures indicated the adequacy and relevancy of EFA procedures. The EFA, using the parallel analysis (PA) test, suggested the presence of four subtle subscale factors that may be extracted from the questionnaire's 23 items. Of which, 6 items were coalesced and loaded saliently (well >0.30) under the first construct (i.e. accreditation policies). Likewise, the remained 17 items were loaded substantively to their relevant intended latent factors and subsequently formed the standards development, evaluation method, and evaluation team constructs. Important to note that none of the items had low loadings or had swayed unmeaningfully under other constructs. Moreover, the tests of closeness-to-unidimensionality indicated that the full questionnaire may not necessarily be a unidimensional scale (UniCo =0.77 which is <0.95, and ECV=0.556 which is <0.85, and M-IREAL score =0.232 which is <0.30). As such, the four subscale scores and the EFA results were accepted because it was simple, meaningful, theoretically sound, and can be interpreted in the context of research. Therefore, EFA results were rotated with the Oblimin method to simplify the analysis.

Furthermore, the robust confirmatory factor analysis (CFA), with the maximum likelihood method assuming polychoric correlations between the questionnaire items, showed a substantial goodness-of-fit between the proposed 4-subscale model. Finally, the 4-subscale factor solution explained a substantial shared covariance between the constructs, which justifies using the mean score for each subscale score by averaging the scores of the items that loaded saliently under each construct. These subscales yield a mean factor-based subscale score bound between 1-5 points for each of them. These mean subscale scores were subject to multivariate linear regression analysis in subsequent statistical analysis as dependent outcome variables.

Table 3
Bivariate Spearman's (Rho) correlations between the perceived importance of accreditation constructs

	AP	SD	EM
Perceived importance of improving accreditation policies (AP)	–		
Perceived importance of improving standards development (SD)	.354**	–	
Perceived importance of improving evaluation methods (EM)	.129	.152	–
Perceived importance of improving evaluation teams (ET)	.071	.003	.060

*** Correlation is significant at 0.010 level.*

Table 4: Multivariate linear regression analysis of the perceived importance of recommended improvements to accreditation policies.

	Standardized β Coefficient	95% CI for β coefficient		p-value
		Lower	Upper	
(Constant)	2.091	1.156	3.027	<0.001
Sex, male	.151	-.084	.385	.206
Educational level, higher education	.285	.068	.503	***.011***
Professional background	-.016	-.066	.034	.529
Having credentials in quality management	.198	-.093	.489	.180
Hospital sector, private	-.078	-.313	.157	.513
Experience in national & international accreditation	.592	.197	.987	***.004***
Years of experience in quality management	.120	.007	.232	***.037***
Perceived importance of standards development	.083	-.083	.250	.325
Perceived importance of evaluation methods	-.184	-.332	-.036	***.015***
Perceived importance of evaluation team	-.104	-.227	.019	.096

Dependent variable: mean perceived importance of the accreditation policies score. Model R=0.641, adjusted R-square=0.327

Table 5: Multivariate linear regression analysis of the perceived importance of recommended improvements to standards development.

	Standardized β Coefficient	95% CI for β coefficient		p-value
		Lower	Upper	
(Constant)	2.716	1.880	3.552	.000
Sex, male	-.016	-.250	.217	.891
Educational level, higher education	-.238	-.454	-.022	***.031***
Professional background	-.029	-.077	.019	.237
Having credentials in quality management	-.008	-.292	.275	.953
Hospital type	.059	-.035	.153	.217
Experience in national & international accreditation	-.171	-.565	.222	.391
Years of experience in quality management	.246	.142	.350	***<0.001***
Perceived importance of accreditation policies	.071	-.088	.230	.379
Perceived importance of evaluation methods	-.023	-.168	.122	.752
Perceived importance of evaluation team	-.159	-.276	-.043	***.008***

Dependent variable: mean perceived importance of standards development score. Model R=0.587, adjusted R-square= 0.299

Table 6: Multivariate linear regression analysis of the perceived importance of recommended improvements to evaluation methods.

	Standardized β Coefficient	95% CI for β coefficient		p-value
		Lower	Upper	
(Constant)	3.576	2.695	4.458	<0.001
Sex, male	-.110	-.362	.141	.388
Professional background	-.034	-.088	.019	.207
Having credentials in quality management	.365	.055	.675	***.021***
Hospital sector, private	-.239	-.490	.012	.062
Experience in national & international accreditation	-.315	-.746	.116	.151
Years of experience in quality management	.268	.154	.381	***<0.001***
Perceived importance of accreditation policies	-.213	-.382	-.044	***.014***
Perceived importance of standards development	-.022	-.201	.156	.805
Perceived importance of evaluation team	-.258	-.385	-.131	***<0.001***

Dependent variable: mean perceived importance of evaluation methods score. Model R=0.558, adjusted R-square=0.269

Table 7: Multivariate linear regression analysis of the perceived importance of recommended improvements to the evaluation team.

	Standardized β Coefficient	95% CI for β coefficient		p-value
		Lower	Upper	
(Constant)	4.898	3.816	5.981	<0.001
Sex, male	-.186	-.495	.124	.238
Professional background	-.049	-.114	.016	.137
Having credentials in quality management	.337	-.041	.716	.080
Hospital sector, private	-.480	-.895	-.065	***.024***
Hospital type	-.124	-.294	.047	.154
Experience in national & international accreditation	-.422	-.944	.100	.112
Years of experience in quality management	.369	.233	.504	***<0.001***
Perceived importance of accreditation policies	-.175	-.383	.033	.099
Perceived importance of standards development	-.255	-.468	-.041	***.020***
Perceived importance of evaluation methods	-.386	-.574	-.199	***<0.001***

Dependent variable: mean perceived importance of evaluation teams score. Model R=0.552, adjusted R-square=0.257

Note on tables 4-7: other variables such as age, clinical experience, and workplace were removed from the above-shown analysis model as they did not correlate significantly with the mean perceived importance of improving the accreditation policies or were found to be redundant (i.e. colinear).
